# Flexible, actin-based ridges colocalise with the *β*1 integrin on the surface of melanoma cells

**DOI:** 10.1038/sj.bjc.6602515

**Published:** 2005-04-20

**Authors:** K Poole, D Müller

**Affiliations:** 1 BioTechnological Center, University of Technology Dresden, Tatzberg 49, D-01307 Dresden, Germany

**Keywords:** melanoma, *β*1 integrin, atomic force microscopy

## Abstract

Using a combination of laser-scanning confocal microscopy and atomic force microscopy, we have identified flexible, actin-based structures on the surface of cells derived from the vertical growth phase of melanoma progression. These flexible structures, lacking on the surface of mature melanocytes, were observed on the surface of all four melanoma cell lines tested. Further investigation revealed that the *β*1 integrin colocalises with these actin-based ridges on the cell surface, whereas *β*1 integrin distribution in melanocytes did not correlate with actin-based structures. Fibronectin staining on the surface of melanoma cells was partially codistributed with the ridges. The combination of structural information derived from atomic force microscopy images and fluorescent imaging of the distribution of labelled proteins involved in invasion and metastasis has allowed us to identify a common feature that may be involved in disease progression, at the surface of vertical growth phase melanoma cells, despite the known variation in genetic composition of melanoma.

The characterisation and study of many disease processes in the past few decades has been dominated by a molecular biological approach, with cells often characterised in terms of varying patterns of gene expression and diseases in terms of genetic mutation. In the case of melanoma, mutations have been found at multiple loci on a number of different chromosomes (Healy *et al*, 1995; Pirker *et al*, 2003). Such diversity results in a complex picture of functional relationships required for the transformation of a melanocyte to a primary melanoma cell. The development of powerful microscopic techniques that can be used in combination allows an alternate approach to the study of such cells, that is, a morphological comparison between cells that may highlight structure/function relationships that are involved in disease progression. Such an approach may be important, as the structural interaction between cells *in situ* influences gene expression and cell function. While electron microscopy allows detailed imaging of treated cell surfaces and cell sections, atomic force microscopy (AFM) can be used to image the surface of cells under physiological conditions (Drake *et al*, 1989). Additionally, AFM can be combined with laser-scanning confocal microscopy (LSCM) to enable comparison of fluorescently labelled surface proteins with surface structures.

The melanoma cell lines imaged here were isolated from tumours in the vertical growth phase (VGP) of development. Such cells display uncontrolled proliferation, metastatic competence and tumorigenicity (Herlyn *et al* 1985, 1987). The VGP primary melanoma invades into the dermis and lesions show increased blood vessel infiltration. Such cells are highly aneuploid, which makes it difficult to characterise them genetically. The VGP cells are known to express a different array of cell surface markers, in comparison to the mature melanocyte (Johnson, 1999). In particular, the expression of integrin adhesion molecules is increased (Natali *et al*, 1993; Duncan *et al*, 1996; Nikkola *et al*, 2004). These integrins have been implicated in the ability of melanoma cells to migrate, invade and metastasise (Nakahara *et al*, 1996, 1998). In this study, we have identified surface structures found on VGP melanoma cells that are not present on the mature melanocyte. These structures were found to be actin based, and to correspond to the localisation of *β*1 integrin, suggesting a role in disease progression.

## MATERIALS AND METHODS

### Cell culture

Melanocytes were obtained from Promocell (Promocell GmbH, Heidelberg, Germany) and cultured in melanocyte growth medium M2, purchased from the same company. Primary melanoma cell lines, WM-39 and WM-853-2 from the Wistar Collection, were a kind gift from Meenhard Herlyn, and were cultured in RPMI 1640 medium containing 5% heat-inactivated fetal calf serum (FCS). Primary melanoma cell lines SK-Mel-28 and WM-115 were from the ATCC collection (LGC Promochem, Middlesex, UK) and cultured in modified MEM medium (ATCC, 30-2003) containing 10% heat-inactivated FCS. Cell cultures were routinely tested for mycoplasma contamination.

### Atomic force microscopy

We conducted AFM imaging using a NanoWizard (JPK Instruments, Berlin, Germany), mounted on a Zeiss Axiovert 200M (Carl Zeiss, Goettingen, Germany). Before imaging, cells were fixed using 2% glutaraldehyde in PBS for 60 s, followed by 3% paraformaldehyde for 20 min. Imaging of cells was performed in PBS containing 1 mM CaCl_2_ and 0.5 mM MgCl_2,_ at room temperature. To compensate for evaporation, the buffer was exchanged at regular intervals of 1 h. Images were taken in low force contact mode using 200-*μ*m-long V-shaped cantilevers, with nominal spring constants of 0.06 N m^−1^. The force applied to the cantilever was adjusted manually to about 50 pN. This force was just sufficient for the stylus of the cantilever to remain in contact with the surface during the scanning process. To optimise image quality, the scan rate was kept between 0.2 and 0.6 Hz. Topographs and error signal images were collected, simultaneously, in both the trace and retrace directions. Heights of topographical features were measured by taking the height at half-maximal width, using the cross-section function contained in the image processing software package from JPK Instruments. All cross-sections were taken parallel to the fast scan axis.

### Laser-scanning confocal microscopy

Cells were grown on 22 × 22 *μ*m coverslips and fixed for 60 s using 2% glutaraldehyde, followed by 3% paraformaldehyde, for 20 min. The membrane was then permeabilised by treating cells for 60 s with 0.5% Triton X-100 in PBS. To label actin, cells were incubated overnight, at 4°C, in PBS containing 1 *μ*g l^−1^ of either FITC- or TRITC-labelled phalloidin (both from Sigma-Aldrich, Munich, Germany). To label *β*1 integrin, cells were incubated with anti-*β*1 integrin antibody (ab3167, Abcam Limited, Cambridge, UK) in PBS, at a dilution of 1 : 500. The secondary antibody was a TRITC-labelled goat anti-mouse IgG antibody (Jackson ImmunoResearch Laboratories, USA), used at a dilution of 1 : 500. Fibronectin was detected by labelling cells with anti-fibronectin antibody used at a dilution of 1 : 300 (Sigma-Aldrich). The secondary antibody was a TRITC-labelled goat anti-rabbit IgG antibody (Jackson ImmunoResearch Laboratories, USA) used at a dilution of 1 : 300. Cells were imaged in PBS with an LSM 510 Meta (Carl Zeiss, Jena, Germany) using a × 63, 1.4 NA oil immersion objective. All confocal images of dual-labelled samples were taken as single track to avoid crosstalk.

### Adhesion assay

Adhesion assays were conducted essentially as described by Lewis *et al* (1996). Briefly, 24-well tissue culture plates were coated with fibronectin (50 *μ*g ml^−1^) overnight in PBS, pH 7.4. Before use, wells were blocked with 10 mg ml^−1^ heat-denatured BSA. Cells were detached with trypsin and washed twice in the relevant growth media, containing trypsin inhibitor. After resuspension, 1 × 10^4^ cells were added to each well. After 1 h, the cells were washed once with medium and three times with PBS. The remaining adherent cells in each well were incubated with 200 *μ*l of solution containing 50 mM sodium acetate, pH 5.0, 0.4% Triton X-100 and 3 mg ml^−1^ nitrophenylphosphate (N4645, Sigma-Aldrich) for 1 h at room temperature. Subsequently, 50 *μ*l of 1 M NaOH was added to each well and samples were then transferred to a 96-well plate and the absorbance at 405 nm measured. The absorbance measured for each sample was compared to the absorbance obtained when the assay was conducted on a sample containing 10^4^ cells, for each relevant cell line, and expressed as a percentage.

## RESULTS

**Figure 1:**
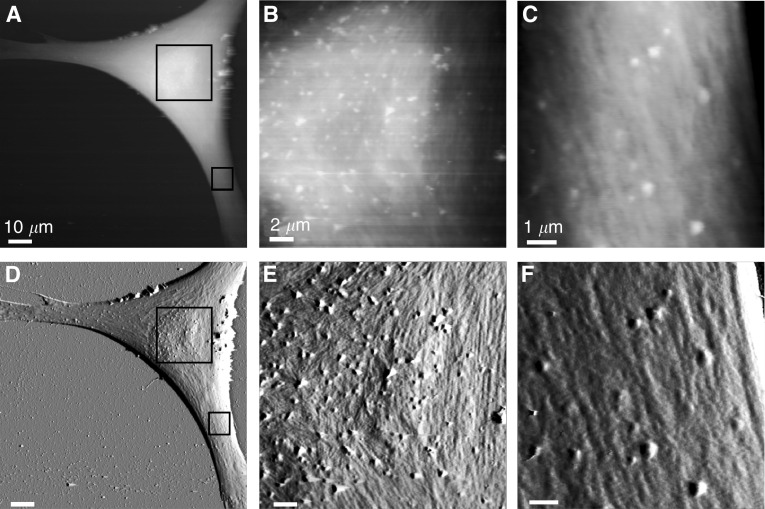
Melanocyte surfaces imaged by AFM. The surface structure of human melanocytes, visualised as AFM topographs (**A**–**C**) and corresponding error signal images (**D**–**F**). Overview images (**A**, **D**) reveal the spindle structure of the melanocytes. Higher magnification images (**B**, **C**, **E**, **F**) reveal subsurface striations corresponding to the cytoskeleton and stable protrusions. Topograph height ranges correspond to (**A**) 6.5 *μ*m, (**B**) 2.1 *μ*m and (**C**) 2.2 *μ*m. All images presented were recorded in the trace scan direction.

The AFM images of healthy, human melanocytes show that the cell surface is relatively devoid of surface structure (Figure 1). Melanocytes are derived from neural crest cells, and maintain long dendrites. In the AFM image, striations in the surface topography running from the cell body to the ends of the dendrites are observed. It has previously been determined that such structures in AFM topographs correspond to the subsurface actin cytoskeleton (Henderson *et al*, 1992; Pietrasanta *et al*, 1994; Braet *et al*, 2001). One also notes a number of spherical protrusions (average height: 83±29 nm, *n*=50), mostly clustered over the cell body, but also along the dendrites. These protrusions are not displaced during the scanning process, despite the movement of the AFM stylus across the cell surface.

**Figure 2:**
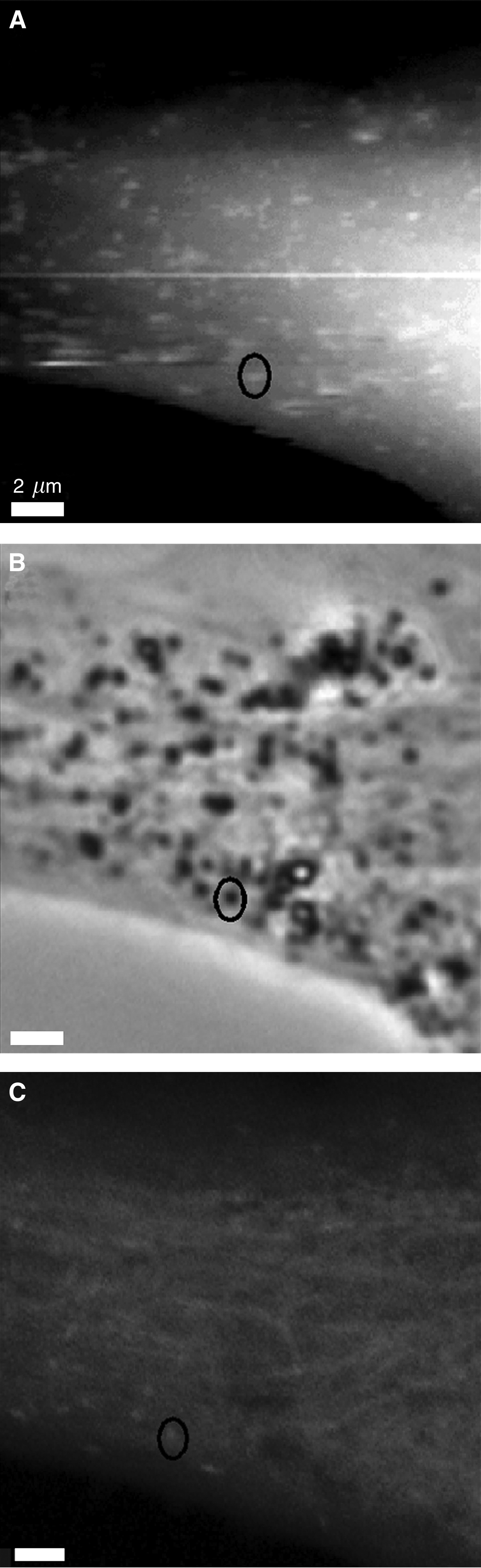
The protrusions on the surface of melanocytes correspond to actin-associated vesicles. The AFM topograph (**A**; height range: 4.3 *μ*m) and corresponding phase-contrast image (**B**) of melanocytes treated with FITC-phalloidin show that some of the protrusions observed in the AFM topograph correspond to vesicles. Additionally, the LSCM image (**C**; optical slice: 0.8 *μ*m) shows brighter actin staining at a fraction of the corresponding points. The AFM image was recorded in the trace scan direction.

The mechanical interaction of the AFM stylus with a sample can lead to dominance in the resulting images of features that have increased stiffness. Although there is currently no effective way of labelling specific structures for detection using AFM, the AFM images can be compared with corresponding light microscopy images, in an effort to characterise specific features. As such, to determine whether the protrusions on the surface of the melanocytes correspond to subsurface vesicles or actin-based structures, filamentous actin was labelled using FITC-phalloidin and cells were imaged by phase-contrast microscopy, LSCM and AFM (Figure 2). The use of LSCM, as opposed to epifluorescence, was important such that fluorescent structures just below cell surface could be compared with the AFM images, as AFM is a surface technique. It can be seen that some of the protrusions detected using AFM correspond to vesicles in the phase-contrast image. Interestingly, filamentous actin is also stained at some of these points. As such, the protrusions observed in the AFM image cannot be attributed to a single macromolecular structure.

**Figure 3:**
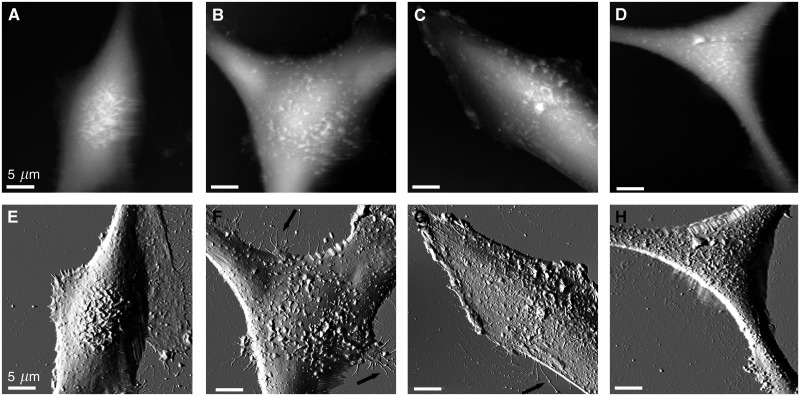
Melanoma cell surfaces from VGP tumours are characterised by highly flexible ridges. The AFM topographs (**A**–**D**) and corresponding error signal images (**E**–**H**) reveal flexible ridges on the surface of four distinct melanoma cell lines, SK-Mel-28 (**A**, **E**), WM-115 (**B**, **E**), WM-853-2 (**C**, **G**) and WM-39 (**D**, **H**). Arrows indicate filopodial extensions. Height ranges correspond to (**A**) 8.7 *μ*m, (**B**) 8.4 *μ*m, (**C**) 6.9 *μ*m and (**D**) 7.8 *μ*m. Images presented were recorded in the trace scan direction.

Imaging of four VGP melanoma cell lines with the AFM revealed distinct differences in surface structure, in comparison with the healthy melanocytes. Flexible ridges were observed on the surface of all cells imaged (Figure 3). The height of these ridges was highly variable (SK-Mel-28=332±156 nm, WM-115=305±156 nm, WM-853=319±192 nm, WM-39=270±179 nm), yet significantly larger than the spherical protrusions noted on the surface of melanocytes. As the AFM tip interacts with the surface of the cell during scanning (albeit at a low force), these flexible ridges are displaced in the direction of the scan, which would also lead to an underestimation of the height of these structures. In some cases, numerous philopodia were observed, extending from the dendrites. To ensure that the observed differences in surface structure did not arise as a consequence of culture conditions, the melanoma cell lines were imaged after culture through three passages in defined M2 media. All cell lines retained the actin-based surface structures (data not shown).

**Figure 4:**
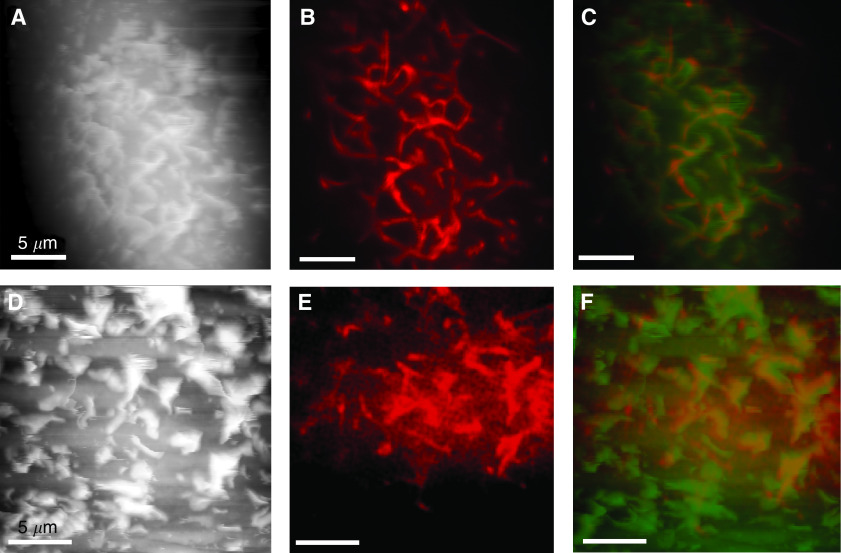
Flexible structures on the surface of VGP melanoma cells are actin based. The AFM topographs (height ranges: (**A**) 6.7 *μ*m; (**D**) 6.7 *μ*m) and LSCM images (**B**, **E**; optical slice: 0.8 *μ*m) of cells treated with TRITC-labelled phalloidin reveal that the flexible structures on the surface of VGP melanoma cells are actin based, in cell lines SK-Mel-28 (**A**–**C**) and WM-115 (**D**–**F**). Overlay images (**C**, **F**), with the AFM topograph in false green and confocal images in red, highlight the correlation. The AFM topographs presented were recorded in the trace scan direction.

Previous studies have also shown that flexible surface structures imaged with the AFM may be actin based (Poole *et al*, 2004). This led us to determine whether the flexible ridges on the surface of the primary melanocyte cultures were actin based. We selected two of the primary melanoma cell lines, SK-Mel-28 and WM-115, for further study and labelled filamentous actin with TRITC-phalloidin. The cells were subsequently imaged with both AFM and LSCM (Figure 4). Comparison of structures in the two images was hindered slightly by the displacement of the flexible ridges in the scan direction by the AFM stylus. However, it is clear that these ridges are actin-based structures.

**Figure 5:**
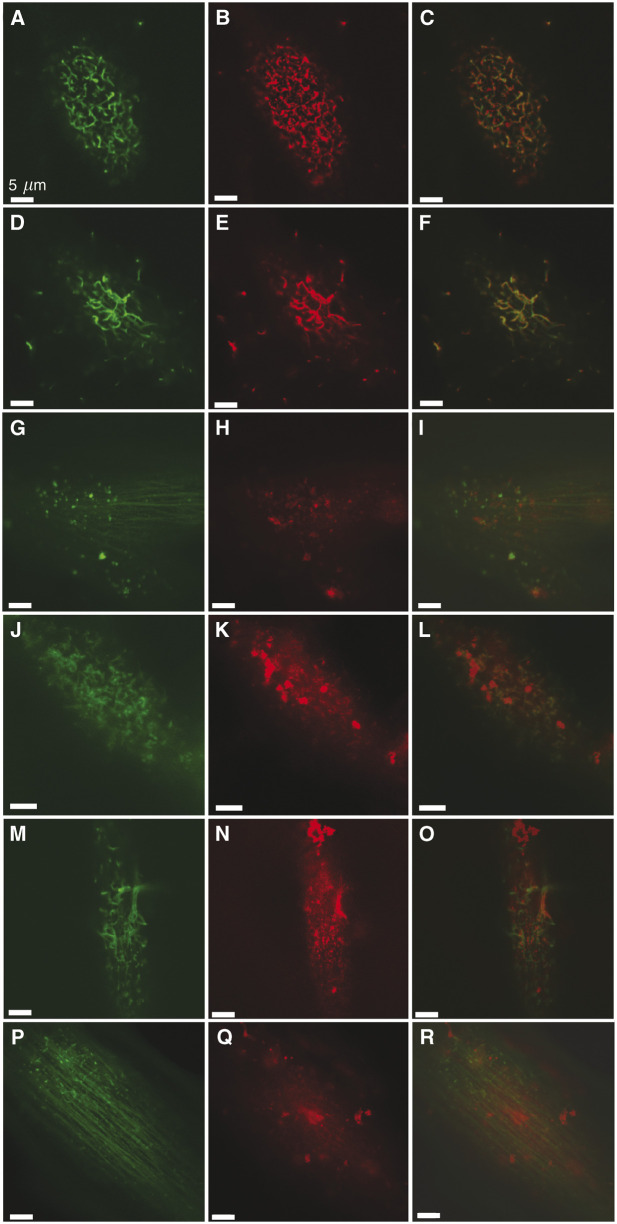
The *β*1 integrin colocalises with subsurface actin structures. The LSCM images (optical slice: 0.8 *μ*m) of SK-Mel-28 (**A**–**C**) and WM-115 (**D**–**F**) cells, dual labelled with FITC-phalloidin (**A**, **D**) and anti-*β*1 integrin antibody, detected using TRITC-conjugated goat anti-mouse secondary antibody (**B**, **E**), reveal that the *β*1 integrin distribution correlates with the subsurface actin structures (overlays: **C**, **F**). The *β*1 integrin was more diffusely distributed on the surface of melanocytes, with some association with actin-associated protrusions noted (labelled actin (**G**), labelled fibronectin (**H**), overlay (**I**)). The LSCM images of SK-Mel-28 (**J**–**L**) and WM-115 (**M**–**O**) cells dual labelled with FITC-phalloidin (**J**, **M**) and anti-fibronectin antibody detected using TRITC-conjugated secondary antibody (**K**, **N**) show that fibronectin is partially bound at the actin-based ridges (overlays: **L**, **O**). In comparison, the LSCM images of melanocytes (**P**–**R**) dual labeled with FITC-phalloidin (**P**) and anti-fibronectin detected using TRITC conjugated antibody (**Q**) do not reveal a correlation between actin-based surface structures and bound fibronectic (overlay, **R**).

Integrin receptors have an important role in cell attachment to the extracellular matrix, signal transduction postattachment and in organising the actin cytoskeleton. In view of this, healthy melanocytes and the two primary melanoma cell lines SK-Mel-28 and WM-115 were analysed to determine localisation of *β*1 integrin on the surface of the cell. Cells were fixed and labelled with both FITC-labelled phalloidin and anti-*β*1 integrin antibodies. The surface of these cells was then imaged using LSCM. It can clearly be seen that the distribution of *β*1 integrin corresponds to the actin-based ridges at the surface of the cells, in both melanoma cell lines. In comparison, *β*1 integrin was distributed over the surface of healthy melanocytes. Punctate staining of *β*1 integrin was observed; however these patches of brighter *β*1 integrin staining were not observed at the same position as the actin-based structures (Figure 5).

The *β*1 integrin is known to be involved in binding to fibronectin. To determine if fibronectin binds to the ridge-associated *β*1 integrin, cells were treated with FITC-phalloidin to label filamentous actin, and anti-fibronectin antibody before imaging with LSCM (Figure 5). Both melanoma cell lines, SK-Mel-28 and WM-115, had surface-associated fibronectin. The fibronectin bound to some of the actin-based ridges, however, not exclusively and not all of the ridges were covered in fibronectin (Figure 5). Additionally, the ability of cells to bind to fibronectin was analysed using an adhesion assay. Cell culture wells were coated with fibronectin, and the percentage adherence of melanocytes, SK-Mel-28 and WM-115 cells was measured after 1 h. The adherence of melanocytes within this time frame was undetectable by this assay for the SK-Mel-28 cell line 32±6% and for the WM-115 cell line 68±7%.

## DISCUSSION

We have identified a common structural feature on the surface of four melanoma cell lines isolated from VGP lesions. The further investigation of two of these cell lines revealed that these flexible structures are actin based. Actin-based dorsal ruffles have previously been identified on cells transformed with Rous sarcoma virus (Marchisio *et al*, 1984). As such, we suggest that these actin-based ridges are a result of melanocyte transformation. In addition, the *β*1 integrin associates with these protrusions, where fibronectin also binds. The *β*1 integrin was also present on the surface of melanocytes; however, the distribution of the *β*1 integrin did not correlate with the smaller actin-based protrusions on this cell line. Additionally, adherence of melanocytes to fibronectin was significantly lower than the adherence of melanoma cell lines.

The VGP of melanoma progression is characterised by invasion of the dermis, requiring cell surface markers, such as the *β*1 integrin, that allow interaction between melanoma cells and components of the ECM, such as collagen and fibronectin. In human melanocytes, the *β*1 integrin is involved in mediating cell survival signals when cells are bound to fibronectin, in a manner also dependent on the actin cytoskeleton (Scott *et al*, 1997). However, in the SK-Mel-28 cell line, the effect of *β*1 integrin binding on the cell is modified, as these cells were resistant to apoptosis in the absence of *β*1 integrin-mediated cell binding (Scott *et al*, 1997). In fact, in metastatic melanoma, the *β*1 integrin is involved in the regulation of invasion as well as the binding of melanoma to the ECM (Nakahara *et al*, 1996, 1998). This role in invasion has been linked to the association of *β*1 integrin to membrane structures in metastatic cells known as invadopodia (Nakahara *et al*, 1996, 1998; Mueller *et al*, 1999). The cell lines tested here were not from metastases, but from the primary tumour site. However, the WM-115 cell line is known to have produced metastases (Herlyn *et al*, 1985). The structures identified here may correspond to the initial structures required to invade the dermis, through interactions with the ECM. It has been shown that invadopodia are not only involved in binding of cytoskeleton-bound *β*1 integrin to the ECM, but are also associated with increased tyrosine phosphorylation of certain proteins (Mueller and Chen, 1991; Mueller *et al*, 1992). The actin-based ridges, identified here on the surface of VGP melanoma cells, may serve as a platform to increase clustering of certain proteins involved in invasion of the dermis, such as *β*1 integrin and various *β*1 integrin-associated signal transduction molecules. This proposal is supported by the different distribution of the *β*1 integrin, with respect to actin, observed between melanocytes and the VGP-derived melanoma cells.

This study leads to some interesting possibilities. Firstly, the identification of the stage of melanoma progression at which such ridges appear may prove useful as an indicator of metastatic competence and consequent disease-free survival. Secondly, if the appearance of such structures on the surface of melanoma cells does correspond to transformation and metastatic potential, then screening of compounds that disrupt such structures, without otherwise disrupting the cells, may help the design of treatments that are specific for blocking metastasis from the primary tumour site.

Here we have shown that AFM can be used to identify common structures on cells that have significant genetic differences. This may prove useful to help identify facets fundamental to the progression of particular diseases. The use of structural information has been used extensively to study functional properties in single proteins, and this study shows a similar potential for the investigation of cells. Now that AFM can easily be combined with confocal microscopy, mapping of functional proteins with structures over the whole cell surface is possible.
